# How important is distractor efficiency for grading Best Answer Questions?

**DOI:** 10.1186/s12909-020-02463-0

**Published:** 2021-01-07

**Authors:** Thomas Puthiaparampil, Mizanur Rahman

**Affiliations:** 1 Department of Medicine, Faculty of Medicine and Health Sciences, UNIMAS, 94300 Kota Samarahan, Malaysia; 2 Department of Community Medicine and Public Health, Faculty of Medicine and Health Sciences, UNIMAS, 94300 Kota Samarahan, Malaysia

**Keywords:** OBA, BAQ, Distractor efficiency, Functional distractors per item in MCQ

## Abstract

**Background:**

Distractor efficiency and the optimum number of functional distractors per item in One Best Answer Questions have been debated. The prevalence of non-functional distractors has led to a reduction in the number of distractors per item with the advantage of adding more items in the test. The existing literature eludes a definite answer to the question of what distractor efficiency best matches excellent psychometric indices. We examined the relationship between distractor efficiency and the psychometric indices of One Best Answer Questions in search of an answer.

**Methods:**

We analysed 350 items used in 7 professional examinations and determined their distractor efficiency and the number of functional distractors per item. The items were sorted into five groups - excellent, good, fair, remediable and discarded based on their discrimination index. We studied how the distractor efficiency and functional distractors per item correlated with these five groups.

**Results:**

Correlation of distractor efficiency with psychometric indices was significant but far from perfect. The excellent group topped in distractor efficiency in 3 tests, the good group in one test, the remediable group equalled excellent group in one test, and the discarded group topped in 2 tests.

**Conclusions:**

The distractor efficiency did not correlate in a consistent pattern with the discrimination index. Fifty per cent or higher distractor efficiency, not hundred percent, was found to be the optimum.

## Background

One Best Answer (OBA) is synonymously called Best Answer Questions (BAQ) in our institution, because the best answer has to be only one. The commonly used markers of psychometric quality of MCQ are difficulty index (DIFi) and discrimination index (DISi). DIFi is the fraction of examinees who get the answer correct [1–6]. The higher its value, the easier the question. It may range from 1 to 0 (all the examinees to none answering correctly). DISi compares the performance of 27% high-scorers with the performance of 27% low-scorers [5, 6]. The value may range from 1 to -1. One means that all the 27% high-scorers and none of the low-scorers got it correct, while − 1 means the reverse. A third metric used for the quality of BAQ is distractor efficiency (DE). Functional distractors per item (FDs/item) and DE in percentage are used to indicate how well the distractors performed their function of distracting the low-scorers. We used both these metrics, because six of our tests contained 5-option items and one test 4-option items. For practical purposes, a distractor is considered functional, if it is chosen as the answer by at least 5% of the examinees [1, 6–8]. Some studies, for example Gajjar et al., used 1% uptake to define functional distractors [9]. This would increase the number of FDs/item and the DE further. Our literature search did not provide a definite answer to our research question of what is the relationship between DE and the quality of items determined by DISi. Lately, the number of options in BAQ items has dropped from 5 to 4 or 3, and many authors considered 3-option BAQ more appropriate, as more items could be included in the test instead of lengthening the tests with non-functioning distractors, as NFDs did not serve any useful purpose [5, 6, 8, 10]. The notion that, the more the FDs/item the better the quality of the item, has also been disputed [1, 4, 11]. It is natural to presume that the highest quality items should have excellent DISi, optimum DIFi and cent percent DE. The null hypothesis of this study was ‘the distractor efficiency or FDs/item in BAQ will correlate directly with the quality of the items determined by their DISi’.

## Methods

### Setting

This was a retrospective study, which analysed 350 BAQs, 50 items per test, used in 7 final professional medical examinations (FPE) of the academic years 2013-14 to 2018-19. Each year our faculty conducts an FPE and a supplementary examination, which employs 6 assessments: (MCQ (60), BAQ (50), MEQ (5), OSCE (20), long case (1) and short cases (3). All the questions were newly written and vetted at the department and faculty levels. Clinical examinations were conducted on real patients. This study used the BAQ papers and their OMR item analysis for DIFi, DISi and number of FDs/item. The DE was calculated manually. The only criterion used for selection of BAQ papers was their availability. There were no changes in the quality of students, curriculum and the assessments, except the lowering of BAQ options from 5 to 4 in test 7. All the data used in this study belong to the Faculty of Medicine and Health Sciences of a public university in Malaysia. Permission was obtained from the dean for utilising the data.

### Data capture and analysis

Most studies used non-functional distractors (NFD) to calculate DE in a round-about manner. We used the number of FDs/item and DE derived from them to make it less confusing. We classified each test of 50 questions, as well as the 350 items put together, into 5 groups according to their DISi value. The data were entered into an Excel Worksheet and the items sorted according to their DISi values from the highest to the lowest along with their DIFi and the number of FDs/item and DE in percentage. Some adjustments were necessary to accommodate all items with varying DIFi values, but they did not affect the order of the groups determined by DISi values of the items. The groups were: Excellent (A) (DISi ≥ 0.40 and DIFi 0.41–0.60); Good (B) (DISi 0.39 − 0.15 and DIFi as in group A or DISi ≥ 0.15 and DIFi 0.31–0.40 or 0.61–0.79); Fair (C) (DISi ≥ 0.15 and DIFi ≤ 0.30 or ≥ 0.80; Remediable (D) (DISi 0.01–0.14 and any DIFi) and Discarded (E) (DISi ≤ 0 and any DIFi). As 6 tests were 5-option type and 1 test 4-option type, we used FDs/item and DE in percentage for the calculations: items with 0 FD (0 DE), 1 out of 4 FD (25% DE), 2 out of 4 FD (50% DE), 3 out of 4 FD (75% DE), 4 out of 4 FD (100% DE) and 2 out of 3 FD (> 50% DE). The mean FDs/item and mean DE of all the 5 groups in 7 tests were compared test-wise and the whole lot combined (Tables 1, 2 and 3).

**Table 1:** Particulars and psychometrics of the 7 BAQ tests with 50 items each

Test	Year	St n	Type	Mean DIFi (SD)	Mean DISi (SD)	Items with 100% DE	Items with DE of ≥ 50%	Mean FD (SD)
1	2013-14	87	FPE 5-op	0.60 (0.25)	0.22 (0.16)	6 (12%)	32 (64%)	2.1 (1.07)
2	2014-15	121	FPE 5-op	0.65 (0.23)	0.22 (0.16)	6 (12%)	34 (68%)	1.96 (1.20)
3	2015-16	125	FPE 5-op	0.69 (0.25)	0.17 (0.14)	5 (10%)	30 (60%)	1.94 (1.24)
4	2016-17	112	FPE 5-op	0.53 (0.28)	0.14 (0.13)	8 (16%)	34 (68%)	2.16 (1.2)
5	2016-17	24	Sup 5-op	0.54 (0.25)	0.16 (0.20)	1 (2%)	30 (60%)	1.72 (0.95)
6	2017-18	118	FPE 5-op	0.63 (0.28)	0.18 (0.15)	3 (6%)	27 (54%)	1.72 (1.23)
7	2018-19	122	FPE 4-op	0.69 (0.23)	0.12 (0.12)	8 (16%)	25 (50%)	1.48 (0.97)

*FPE *final professional examination, *op *options, *DE *distractor efficiency, *FD *functional distractors per item, *DIFi *difficulty index, *DISi *discrimination index, *St *students, *n *number. Items with ≥ 50% DE include items with 100% DE

**Table 2:** BAQ items sorted into 5 classes showing their psychometric properties

Classes	Mean DIFi (SD) and range	Mean DISi (SD) range	n and (% of items)	Mean FDs/item (SD) and range
A (excellent)	0.53 (0.05) 0.43–0.59	0.47 (0.07) 0.4–0.59	9 (2.57)	2.44 (0.53) 2–3
B (good)	0.58 (0.14) 0.31–0.79	0.3 (0.1) 0.15–0.64	122 (34.86)	2.3 (0.91) 1–4
C (fair)	0.6 (0.31) 0.08–0.93	0.23 (0.07) 0.15–0.50	61 (17.43)	2.07 (1.14) 0–4
D (remediable)	0.68 (0.29) 0.04–0.99	0.08 (0.04) 0.01–0.14	90 (25.71)	1.42 (1.19) 0–4
E (discarded)	0.61 (0.33) 0–1	-0.02 (0.05) -0.20–0	68 (19.43)	1.45 (1.17) 0–4
	TOTAL		350 (100)	654/350 = 1.87

*BAQ *best answer questions,* DIFi* difficulty index, *DISi *discrimination index, *DE *distractor efficiency, *FD *functional distractors

**Table 3:** BAQ tests sorted into 5 classes or groups showing their distractor efficiency

Test	A n	A DE (%)	B n	B DE (%)	C n	C DE (%)	D n	D DE (%)	E n	E DE (%)	Mean DE (%)
1	2	62.5	19	57.89	13	48.08	10	37.5	6	66.67	52.5
2	1	50	24	64.58	10	47.5	13	17.31	2	75	49
3	1	75	16	65.63	13	51.92	15	23.33	5	55	48.5
4	1	50	12	56.25	8	53.13	18	52.78	11	40.91	54
5	1	75	17	48.53	5	65	8	34.38	19	34.21	43
6	2	62.5	19	57.89	6	37.5	17	33.82	6	12.5	43
7	1	66.66	15	62.22	6	55.55	9	66.66	19	28.07	49.33
Mean		63.09		59		44.1		37.97		44.62	

Class of items: A = excellent, B = good, C = acceptable, D = remediable, E = discarded

*DE *distractor efficiency in percentage,* n *number of items, classes A, B and C were banked, D put in remediable category and E discarded

## Results

### The psychometrics of the 7 BAQ tests

The mean DIFi ranged from 0.53 to 0.69, mean DISi from 0.12 to 0.22, items with 100% DE from 2–16%, items with 50% and above DE from 50–68% and the mean FDs/item from 1.48 to 2.16.

### The psychometrics of the 350 BAQ items sorted into 5 classes

Excellent items (A) were 2.57% of the 350 items, and they had the highest mean FDs/item of 2.44 (range 2–3). Good items (B) were 34.86% with the second best mean FDs/item of 2.3 (range 1–4). Fair items (C) were 17.43% and had a mean FDs/item of 2.07 (range 0–4). Remediable items (D) were 25.71% and had a mean FDs/item of 1.42 (range 0–4). The discarded items (E), which had 0 or negative DISi, were 19.43% and had a higher FDs/item of 1.45 (range 0–4) than the remediable items (Table 2).

The 350 items of 7 tests were sorted into 5 grades based on DISi. The mean DE of all grades and the overall mean DE of the tests were calculated.

**Fig. 1:**
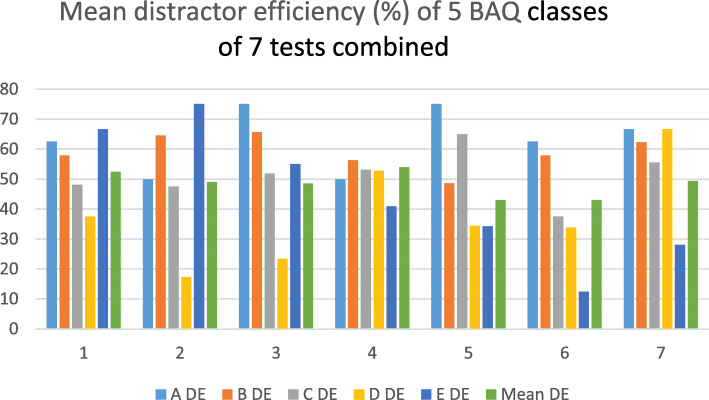
Mean distractor efficiency of 5 classes of BAQ and the overall mean DE of 7 tests

Test 4, with the highest number of D and E items combined, had the highest mean DE among the 7 tests. In tests 1 and 2, class E had the highest mean DE. class B had higher mean DE than class A in tests 2 and 4 (Table 3). Class A had the highest mean DE in tests 3 and 5, B in test 3, C in test 5, D in test 7, and E in test 2. Mean DE was highest in test 4 and lowest in tests 5 and 6 (Table 3; Fig. 1).

### The correlation coefficients of DIFi and DISi with mean FDs/item in the 350 BAQs

DIFi with FDs/item showed significant negative correlation, which meant the easier the item, the less the number of FDs/item, while DISi with FD showed a significant positive correlation, which meant the higher the number of FDs/item, the higher the DISi (Table 4)

**Table 4:** Correlation of DIFi with FDs/item and DISi with FDs/item of 350 BAQ items

	DIFi - FD	DISi - FD	
Pearson correlation r	− 0.66 (Sig. 0.000)	0.317 (Sig. 0.000)	
Spearman’s rho	− 0.705 (Sig. 0.000)	0.347 (Sig. 000)	

Correlation is significant at the 0.01 level (2-tailed)

*DIFi* difficulty index; *DISi* discrimination index; *FD  *functional distractors per item

## Discussion

The psychometrics of BAQ tests reflect the quality of the items as well as the quality of the examinees, and therefore, they cannot be taken as absolute values. Score distribution curve and standard error of measurement are important to make further judgements about the tests [5]. Our study did not perform such quality checks. The performance of our tests in terms of psychometrics was comparable with that of other studies. The proportion of reusable items (the same as A, B and C groups of our study combined) ranged from 80% [11], 60% [12], 46.66% [7] to 30% compared to 54.66% in this study. Items with ‘0’ DE ranged from 26%, 23% [6, 11], 12.3% [4], 2–0% [7, 12] compared to 12.29% in this study. Items with 50% and above DE ranged from 90% [7], 85% [12], 63.33% [11], 50% [13], 47.5% [4], 39.1% [4] to 13% compared to 60.57% in this study and items with 100% DE ranged from 70%, 56.67% [7], 25% [12, 13], 13.8% [4], 6.66% [11] to 2.9% [6] compared to 10.29% in this study. The mean FDs/item in our tests ranged from 1.48 to 2.16, with a mean of 1.87 (Table 1), which was also comparable [14]. Among the variables we used, test 5 was an outlier, as it had only 24 students. However, since its psychometrics were at par with those of the other tests, we did not drop it from the study. The outstanding finding of our study was that there was no consistent pattern, as to which group had the highest or the lowest DE. The mean DE of some lower groups surpassed those of higher groups. DE of group E was the highest in tests 1 and 2; higher than those of groups C and D in test 3; higher in B than in A in tests 2 and 4; and D being higher than B and C and equal to A in test 7 (Table 3). These findings supported the rejection of our null hypothesis. Our study showed that the DE in the excellent group ranged from 75–50% and in the discarded group ranged from 75–12.5% (Table 3). It was interesting to note that FDs/item ranged from 2 to 3 in group A, 1 to 4 in group B and from 0 to 4 in groups C, D and E (Table 2; Fig. 1). This supported our conclusion that 4 FD or 100% DE was not particular to the high-class items. Many FDs but negative DISi means, they are not really FDs, as they distracted the high-scorers more than the low-scorers. The discarded items with 0 or negative DISi had higher mean FDs/item than the remediable category. Moreover, the groups lower than A had a higher frequency of 100% DE (Table 2). The overall correlation coefficients of DIFi and DISi with mean FDs per item in BAQ were far from perfect. Our study showed that tests with the highest number of reusable items (A, B, C) (test 2 with 70% and test 1 with 68%) had a mean FDs/item of 1.96 (1.2 SD) and 2.1 (1.07 SD), while test 4 with the lowest number of reusable items (42%) had a mean FDs/item of 2.16 (1.2 SD) (Tables 1 and 3).

Some authors have claimed that DE would influence other psychometrics indices, and so it needs to be considered while selecting items for reuse [7, 11]. If 5% examinees each chose the 4 distractors (5 × 4 = 20%), the item would have 100% DE and 0.8 DIFi. It is possible to have a single FD in a difficult, optimal or easy item. Similarly, 2 FD, 3 FD or 4 FD items might have very high or low DIFi. It meant DE and DIFi do not have a predictable relationship. Some studies support this view [1, 4]. What about the relationship of DISi with DE? The highest DISi of + 1 means 27% high scorers got the item correct and 27% low-scorers, who chose the distractors, went wrong. The remaining 46% could have chosen either way. So, even if 73% of examinees have gone wrong (DIFi 0.27), or right (DIFi 0.73), the item could have a DISi of + 1 and DE of 100%. If only 27% low-scorers got the answer correct, the item could have a DIFi of 0.27, DISi of -1 and DE of 100%. It shows that DE does not have a predictable influence on DISi, as shown in our results, which is supported by the literature [13, 14]. Hassan et al. [11] argued that low DE lowers DISi [11]. Hingorjo and Jaleel [3] reported similar findings whereby items with 1 NFD were better than those with none [3]. The items with poor and good DISi had almost the same DE, and that DE in items with high and low DIFi was the same [13]. Licona-Chávez et al. [12] analysed DIFi, DISi, DE and Cronbach alpha to evaluate 20 MCQ items, but did not find a parallel performance in all four metrics. Items with 100% DE did not gain excellent DISi [12]. Reducing the number of distractors in items did not affect DISi significantly [14]. DIFi with FDs/item showed a significant negative correlation, and DISi with FDs/item showed a significant positive correlation, but both were not highly significant (Table 4).

Difficulty experienced in constructing plausible distractors led to a recommendation of 3-option items [4, 8, 13, 15]. The reliability and validity of tests improve with more items allowing more comprehensive content coverage in tests [5]. As NFDs do not serve their function, dropping them to create fewer option items allowing wider content coverage is worthwhile. Therefore, based on the findings from this study, we suggest that 2 or more FDs per item or 50% or higher DE as optimum. Finally, although psychometrics are important, expert review of questions is obligatory. We recommend an expert review of all items despite excellent to acceptable psychometric performance. Our recommendation is consistent with that of McCoubrie [16].

### Limitations

We could not analyse the tests for student scores distribution, standard error of measurement and reliability coefficients due to unavailability of some test scores. The 5-option tests were 6 while the 4-option test was only one.

## Conclusions

Our study, as several other studies, showed the importance of using DISi, DIFi and DE in evaluating items and selecting them for reuse. We could reject the null hypothesis as the DE and FDs/item did not go in parallel to the classes of the items. No definite pattern of DE could be seen in correlation with excellent, good or rejected items in our study. Items having the highest DE were not the best in quality by the DISi standards. It was a mixed pattern with some surprises like discarded items having higher DE in some tests. We conclude that, although DE is important for BAQ, items with 100% DE are not the best, when other psychometrics are considered. A DE of 50% or above should be taken as optimum. Also, psychometrics should not replace expert review of items.

## Data Availability

The data will not be available publicly. The principal author keeps all the materials and data used in this study.

## Abbreviations

BAQ: Best Answer Question
DE: Distractor efficiency
DIFi: Difficulty index
DISi: Discrimination index
FD: Functional distractor
IRB: Institutional Review Board
MCQ: Multiple Choice Question
NFD: Non-functional distractor
OBA: One best answer question
SD: Standard deviation
UNIMAS: Universiti Malaysia Sarawak
